# Chiral Selectors in Capillary Electrophoresis: Trends during 2017–2018

**DOI:** 10.3390/molecules24061135

**Published:** 2019-03-21

**Authors:** Raymond B. Yu, Joselito P. Quirino

**Affiliations:** Australian Centre for Research on Separation Science (ACROSS), School of Natural Sciences-Chemistry, University of Tasmania, Hobart, 7001 Tasmania, Australia; raymond.yu@utas.edu.au

**Keywords:** chiral separation, capillary electrophoresis, electrokinetic chromatography, macromolecules, supramolecules

## Abstract

Chiral separation is an important process in the chemical and pharmaceutical industries. From the analytical chemistry perspective, chiral separation is required for assessing the fit-for-purpose and the safety of chemical products. Capillary electrophoresis, in the electrokinetic chromatography mode is an established analytical technique for chiral separations. A water-soluble chiral selector is typically used. This review therefore examines the use of various chiral selectors in electrokinetic chromatography during 2017–2018. The chiral selectors were both low and high (macromolecules) molecular mass molecules as well as molecular aggregates (supramolecules). There were 58 papers found by search in Scopus, indicating continuous and active activity in this research area. The macromolecules were sugar-, amino acid-, and nucleic acid-based polymers. The supramolecules were bile salt micelles. The low molecular mass selectors were mainly ionic liquids and complexes with a central ion. A majority of the papers were on the use or preparation of sugar-based macromolecules, e.g., native or derivatised cyclodextrins. Studies to explain chiral recognition of macromolecular and supramolecular chiral selectors were mainly done by molecular modelling and nuclear magnetic resonance spectroscopy. Demonstrations were predominantly on drug analysis for the separation of racemates.

## 1. Introduction

Bioactive chiral compounds are structurally similar, but they can exhibit different biological activity and even potency [1,2]. A classic example is thalidomide, which has a therapeutic *R*-isomer and a teratogenic *S*-isomer. Analytical chiral separation is therefore important for these compounds, e.g., for purity or enantiomeric excess assessment of active or in-process chemicals in the pharmaceutical and pesticide industries. Analytical chiral separation is typically performed using column chromatography-based methods such as liquid chromatography (LC) or gas chromatography (GC) with a chiral stationary phase [3,4,5] and capillary electrophoresis (CE) with a chiral pseudophase [6,7,8]. The CE mode used for chiral separation is known as electrokinetic chromatography (EKC) [9]. A chiral selector (CS) is embedded in the stationary phase or pseudophase, and more often in EKC the CS is the pseudophase. The CS is the chiral component of the separation system that interacts enantioselectively with the targeted enantiomers. This interaction causes the separation of enantiomers. Host-guest complexes are formed via non-covalent interactions such as dipole, electrostatic, hydrogen bonding and steric interactions.

**Figure 1:**
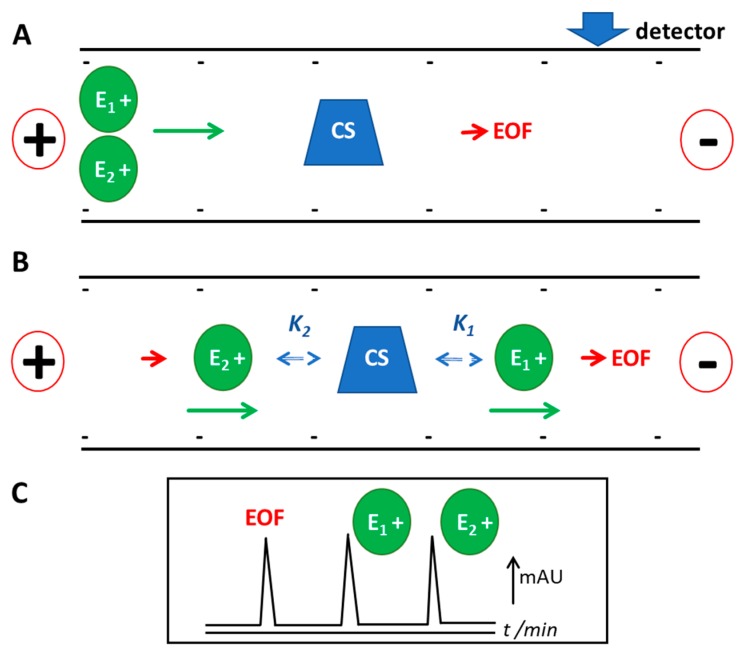
Chiral separation of a positively charged racemate in EKC with a neutral CS as pseudophase. The initial condition is shown in (**A**). The fused silica capillary contains (negative) ionisable silanol groups at the walls. The capillary is filled with background solution (typically a buffer to maintain a certain pH) that contains CS. The sample (containing the two enantiomers) is injected in the inlet or left end of the capillary. A voltage is applied at positive polarity, or negative electrode at the outlet or detector end. The electric field produced drives the separation and detection of the analytes. The direction of EOF and electrophoretic velocity of analytes are to the cathode (right, detector end). The neutral CS migrates in the same velocity as the EOF. The interaction of enantiomer 2 (E_2_) to the neutral CS is stronger compared to enantiomer 1 (E_1_), as depicted in (**B**). The binding constant (K) for E_2_ is larger compared to E_1_, or K_2_ > K_1_. The apparent velocity of E_2_ (in the direction of the cathode) is slower than E_1_. E_1_ is thus detected earlier than E_2_, as shown in the drawn electrochromatogram in (**C**). The EOF (with a neutral probe) is detected after the positively charged enantiomers. The difference in apparent velocity due to the differential interaction of the enantiomers with the pseudophase causes the chiral separation.

One of the successful applications of CE is in analytical chiral separation. EKC is a nano-/micro- scale technique and thus it inherently supports green analytical chemistry [10]. The amounts of samples and consumables required are very low and chemical waste generation is essentially negligible. In comparison with LC EKC provides higher separation power (separation efficiencies approaching those of GC). Preparation of a solid chiral stationary phase is also not required since a soluble CS is directly dissolved into the BGS. The instrumental set-up of CE is simple, thus CE experiments are relatively easy to perform. CE uses a narrow inner diameter (i.d.) capillary (e.g., 50 or 75 μm) that is typically 30–80 cm long. The capillary is filled with a background solution (BGS) which functions in the same manner as the mobile phase in LC. The sample is injected at one end of the capillary, and then a voltage is applied with the BGS at both ends of the capillary. The electric field across the capillary causes the electrophoretic migration of analytes and other charged species (e.g., buffer components), and the generation of the bulk liquid flow (i.e., electroosmotic flow (EOF)). In chiral EKC, the migration of the analytes is altered because of the analytes’ interaction with the neutral or charged CS in the BGS. The analytes’ electrophoretic migration and interaction with the pseudophase and EOF eventually cause detection of the analytes at the other end of the capillary. Detection is typically by on-line UV but off-line mass spectrometric detection is also available [11]. A simple schematic of a representative chiral separation by EKC using a fused silica capillary is shown in Figure 1. The general mechanism of separation is discussed in the caption of Figure 1. 

In chiral EKC, CSs can be grouped into: (a) macromolecular (including macrocyclic), (b) supramolecular, and (c) low molecular mass compounds. The pseudophases are classically macromolecules (e.g., cyclodextrins) and supramolecules (e.g., bile salt micelles). Macromolecules are molecules of high relative molecular mass, the structure of which essentially comprise a multiple repetition of units derived, actually or conceptually, from molecules of low relative molecular mass [12]. Supramolecules is a system of two or more molecular entities held together by means of intermolecular non-covalent bonding interactions [12]. They are molecular aggregates.

**Figure 2:**
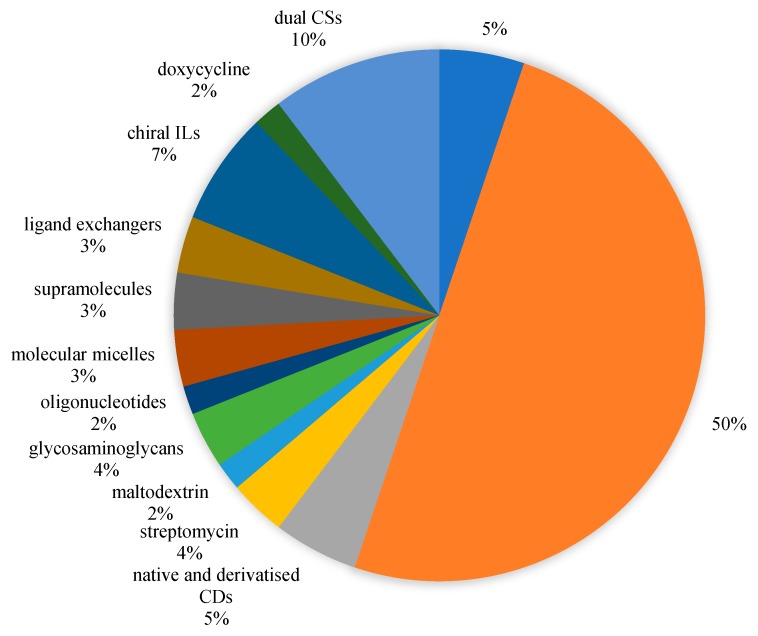
Chiral selectors in EKC during 2017–2018 (58 papers covered).

We found six review articles published on chiral separation in CE during 2017–2018. Chankvetadze discussed the contemporary theories explaining enantioseparations in CE [13]. Stavrou and co-workers covered the utility of various macromolecules from 2014-mid 2016 [14]. Meanwhile, two reviews were specifically on the use of ionic liquids (ILs) [15,16] and one more review that focused on antibiotics as CS [17]. Finally, a review focused on improving UV detection sensitivity of chiral CE methods [18]. The combined topics of macromolecules (part 1), supramolecules (part 2), and low molecular mass molecules (part 3) as CS in chiral EKC from 2017–2018 as done in this review will hopefully provide established and novice researchers a broader picture of recent developments in this field. The search parameters used in Scopus were “cyclodextrins”, “polysaccharides”, “antibiotics”, “bovine serum albumin”, “pepsin”, “oligonucleotides”, “chiral crown ethers”, “polymers”, “molecular micelles”, “micelles” and “chiral separation”. Figure 2 shows the summary of the papers considered. The use of cyclodextrins (CDs) as a single CS or in combination with another CD or CS remains to be popular in EKC, accounting for 67% of all papers. Derivatised CDs as a single CS composed 50% of all papers. This high % could be explained by the high solubility that is required in EKC and better chiral recognition ability of derivatised over native CDs. Five out of the seven papers that implemented dual CSs (10% of all papers) used a CD. Three papers (5% of all papers) evaluated native and derivatised CDs, but not used as dual CSs. The use of a chiral IL as a single CS accounted for 7% of all papers. Its properties such as high solubility and ability to alter the EOF make it an attractive BGS modifier in EKC. Other classes account for 2–4% of the papers covered (streptomycin, polysaccharide-based, nucleotide-based, molecular micelles, supramolecules, ligand exchangers, doxycycline).

## 2. Macromolecules

### 2.1. Sugar-Based Macromolecules

The sugar-based macromolecules considered in this section are the CDs and the polysaccharides maltodextrin and glycosaminoglycans (chondroitin sulphate D and heparin). The pseudo-trisaccharide antibiotic streptomycin is considered under this section.

#### 2.1.1. Cyclodextrins

##### Native CDs

α-, β- and γ-CD are the native CDs, characterized by the increasing size of their hydrophobic cavity. This cavity is responsible for the formation of inclusion complexes with the enantiomers, where one enantiomer could be preferred over the other. α-CD has been the most ignored CS due to its poor selectivity, perhaps due to its small cavity that could limit the relevant host-guest interactions. Improved separations were however reported by the use of α-CD in the presence of chiral ILs (i.e., tetraalkylammonium amino acid chiral ILs) in the BGS in EKC [19], although the claim was not supported by a separation using only the chiral ILs in the BGS. In another study, a separate injection of hydroxypropyl methylcellulose plug in partial filling EKC (PF-EKC) with α-CD in the CS plug improved the separation of d,l-Tyr and d,l-Trp [20]. 

##### Modified CDs

β-CD derivatives, i.e., neutral (hydroxyethyl-, hydroxypropyl- and methyl-) and anionic (carboxymethyl-, and sulphated-) [21,22,23,24,25,26,27,28,29,30,31,32,33] derivatives were the most commonly used CSs reported. Other commercially-available modified CDs or laboratory-prepared modified CDs with known synthesis were reported. These were hydroxypropyl-α-CD [23], sulphated-α-CD [21], (2-carboxy-ethyl)-β-CD [34], heptakis(2,3-di-*O*-acetyl)-β-CD [35], heptakis(2,6-di-*O*-methyl)-β-CD, heptakis-(2,3,6-tri-*O*-methyl)-β-CD [36,37], heptakis(2-*O*-methyl-3,6-di-*O*-sulfo)-β-CD, heptakis(2,3-di-*O*-methyl-6-*O*-sulfo)-β-CD, heptakis(2,3-di-*O*-acetyl-6-*O*-sulfo)-β-CD [36], heptakis-6-sulfato-β-CD [38], heptakis-(6-*O*-sulfobutylether)-β-CD sodium salt [39], heptakis (2,6-di-*O*-[2-hydroxy-3-(sulfo-amino)propoxy])-β-CD [40], 2-hydroxypropyl-β-CD [37] octa(6-*O*-sulfo)-γ-cyclodextrin [41], lysine-bridged hemispherodextrin [42], methylated β-CD [43], mono-6-deoxy-6-(4-amino-1,2,4-triazolium)-β-CD chloride [44], mono-6-deoxy-6-(3-methylimidazolium)-β-CD tosylate [45], quaternary ammonium β-CD [46], carboxymethyl-β-CD [47,48], hydroxypropyl-γ-CDs [21,34], sulphated-γ-CDs [21] and highly sulphated-γ-CDs [49]. Sulfobutylether-β-CDs are also available commercially as Captisol^®^ and were applied successfully in the enantiomeric separation of neutral and basic drugs, but not towards negatively charged analytes in EKC [50].

There were two studies that compared anionic and neutral β-CD derivatives, and both studies concluded that the anionic derivatives provided better selectivity for the analytes tested (cationic higenamine [26] and neutral tramadol [22]). The host-guest interaction with anionic β-CDs are driven by electrostatic interactions, hydrophobic inclusion and hydrogen bonding, which is the case with higenamine. On the other hand, based on the parameters used for computational experiments, tramadol enantiomers that do not fit inside the cavities of anionic carboxymethyl- or sulphated-β-CD were apparently separated by interaction with functional groups outside the cavity. This is in contrast to known mechanisms for chiral recognition with CDs which requires the formation of inclusion complexes driven by specific interactions [51].

An IL-functionalised β-CD, mono-6-deoxy-(4-amino-1,2,4-triazolium)-β-CD chloride was prepared and successfully applied as CS for dansyl amino acids and naproxen [44]. Improved water solubility and additional non-bonding interactions from the IL functionalisation as shown in molecular dynamics calculations were perhaps the reasons behind improved resolution. The IL-functionalised β-CDs were also found to suppress EOF, similar to that observed with a quaternary ammonium β-CD [52]. The cationic groups of these derivatised β-CDs interact with the silanol groups of the capillary wall. The negative charge at the wall is neutralised and the neutral CD part of the derivatised β-CDs is in contact with the bulk solution. 

**Figure 3:**
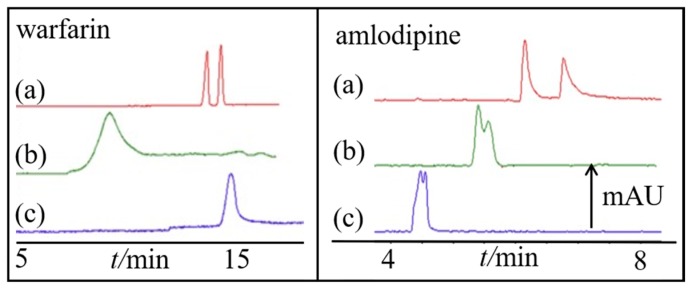
Electrochromatograms under different separation conditions (**a**–**c**). Conditions: fused-silica capillary, 33 cm (24.5 cm effective length) × 50 μm i.d.; capillary temperature, 25 °C; sample injection: 5 s at 50 mbar; For warfarin, applied voltage: (**a**), (**b**) −6 kV, (**c**) 6 kV; BGS: 20 mM phosphate buffered saline (PBS) containing (**a**) 2% SBE-β-CD (*w*/*v*) + 1.2% (*w*/*v*) liposomes, (**b**) 2% SBE-β-CD (*w*/*v*) + 20 mM SDS, (**c**) the single SBE-β-CD system, buffer pH: 8.4; For amlodipine: −10 kV, BGS: 20 mM PBS containing: (**a**) 1.5% SBE-β-CD + 0.96% liposomes; (**b**) 1.5% SBE-β-CD + 16 mM SDS; (**c**) the single SBE-β-CD system; buffer pH 2.6. All the SDS concentrations in the BGS were the same as that of liposomes. Reprinted with permission from [54].

CDs were used as CS in EKC together with non-chiral pseudophases, i.e., micelles [53] and liposomes [54]. The additional pseudophase (non-chiral) could enhance enantioselectivity of CDs and allow additional retention of the analytes. β-CD was evaluated as a CS together with hydroxypropyl-β-CD for the separation of six pairs of d,l-amino acids in micellar EKC (MEKC) with sodium dodecyl sulphate (SDS) [53]. Baseline separation was not achieved with β-CD alone but was improved with hydroxypropyl-β-CD. Meanwhile, liposomal EKC (LEKC) using phosphatidylcholine and cholesterol as pseudophase and sulfobutyl-β-CD (SBE-β-CD) as CS was utilised for the separation of four racemic drugs [54]. Liposomes are vesicles consisting of phospholipid bilayers enclosing an aqueous solution. Figure 3a–c show the separation of warfarin and amlodipine using sulfobutyl-β-CD-LEKC, sulfobutyl-β-CD-MEKC and sulfobutyl-β-CD respectively. In their optimal conditions, sulfobutyl-β-CD-LEKC had better separation compared to the other two modes.

#### 2.1.2. Streptomycin

Streptomycin is an aminoglycoside antibiotic with numerous functional groups together with two hexatomic rings, which allows the interaction with chiral analytes. Streptomycin was a suitable additive to the BGS for the chiral EKC of several drugs due to its low ultraviolet (UV) absorbance [55]. In another study, a dispersion of streptomycin-modified gold nanoparticles as BGS was reported for another set of drugs in EKC [56]. The chiral separation was not proven by the analysis of a pure enantiomer. More importantly, the electrochromatograms presented show no disturbances in the baseline, although gold nanoparticles are known to absorb in the UV region. This physical characteristic allowed the photometric detection of gold nanoparticles [57]. 

#### 2.1.3. Polysaccharides

Polysaccharides contain more than ten monosaccharide residues linked glycosidically. Homopolysaccharides from d-glucose (maltodextrin) and glycosaminoglycans (chondroitin sulphate D and heparin) were used as CSs. Polysaccharides can discriminate enantiomers due to their chirality, emanating from the presence of several stereogenic centers, helical twist of the polymer backbone and alignment of adjacent chains that form ordered regions [58]. 

##### Maltodextrin

Maltodextrin is made up of 2–20 glucose residues linked together by α(1→4) glycosidic bonds and has a helical structure similar to that of amylose. Its high solubility in water makes it a highly suitable additive in EKC. Its helical structure allows it to be a flexible CS, but high concentrations are required for effectiveness. There were three reports where maltodextrin was used alone [59] and in conjunction with other CS [23,60] in EKC. 20% (*w*/*v*) maltodextrin in pH 8.0 BGS was able to separate tramadol and methadone enantiomers satisfactorily [59]. Meanwhile, only 3% (*w*/*v*) maltodextrin but in combination with chiral ILs in the BGS were able to separate four racemic drugs [60]. A comparison of the separations using maltodextrin alone and with 50 mM each of tetramethylammonium hydroxide, tetramethylammonium-d-pantothenate and tetramethylammonium-d-quinate clearly showed an improvement in separation in the presence of an IL. The migration times also increased in the presence of an IL, indicating a role of EOF in the separation; although, the polarity of the applied voltage was not clearly indicated. Interestingly, maltodextrin and CDs (hydroxypropyl-α-CD and hydroxypropyl-β-CD) in the BGS did not act synergistically for chiral separation of basic drugs [23]. However, the implementation of two chiral solution plugs, a maltodextrin and a hydroxypropyl-α-/β-CD plug allowed the separation of the tested drugs in PF-EKC. 

##### Glycosaminoglycans

Chondroitin sulfate D and heparin are highly sulphated heteropolysaccharides. The presence of negative charges increases their water solubility and electrophoretic mobility, making these compounds suitable as BGS additives in EKC. Chondroitin sulfate D is a linear macromolecule made up of alternating d-glucoronic acid and *N*-acetyl-d-glucosamine units, attached by a β(1→4) glycosidic linkage within the disaccharide unit and a β(1→3) glycosidic linkage with another disaccharide unit. Successful separation was claimed to be due to electrostatic, hydrogen bonding, hydrophobic and steric interactions [61]. A study investigated its synergism with carboxymethyl-β-CD using various cationic small molecule drugs as test analytes. The chondroitin sulfate d-carboxymethyl-β-CD selector system was more successful than each individual selector. Attempts to explain the synergism of the two CS using molecular modelling did not yield very clear results [62].

Heparin is a mixture of variably sulphated polysaccharides, whose basic subunit is composed of d-glucosamine and l-iduronic or d-glucuronic acids linked by an α(1→4) glycosidic bond. Unlike chondroitin sulfate D, heparin is a helical molecule. Its three-dimensional structure and negative charge arising from the sulphate groups exposed in the solution contribute to its success as a CS [63]. Improved EKC separation of various drugs were obtained with heparin in the BGS and a 3-aminopropyltriethoxysilane coated capillary [64].

### 2.2. Chiral Selectors Based on Nucleotides as Basic Units

Oligonucleotides and nucleic acids are heteropolymers of nucleotide bases linked together by a sugar-phosphate backbone. Both oligonucleotides and nucleic acids adapt varied three-dimensional structures such as the classical helical structure of double stranded DNA, hairpin structures or G quadruplexes, among others depending on local and external conditions [65]. Oligonucleotides and nucleic acids, due to their water solubility, make them obvious choices as CS additives in EKC. The major issue is the UV activity, thus partial filling techniques are required. The chiral recognition mechanism of oligonucleotides and nucleic acids relied either on classical enantioselective intercalation or groove binding (for double stranded DNA or G4 templates), or enantiospecific allosteric mechanism (for oligonucleotides, e.g. aptamers), making these approaches of limited value in terms of analyte selectivity range and practical applications [66]. However, the high selectivity could be of value for targeted analyses of very important compounds. It has also been noted that earlier studies were focused on oligonucleotides as CS for specific analytes with limited interest [67].

**Figure 4:**
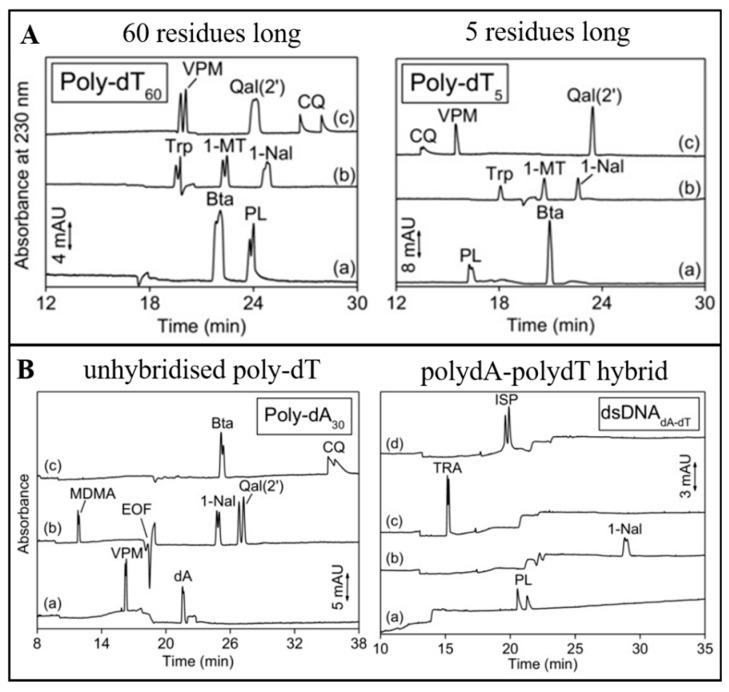
(**A**) Effect of number of oligonucleotide residues (60 vs. five residues) on PF-EKC separation. (**B**) Effect of oligonucleotide hybridisation (unhybridised vs. hybridised) on PF-EKC separation. Conditions: fused silica capillary 105 cm (60 cm to the detector) × 25 μm i.d., applied voltage: +30 kV, temperature: 10 °C, electrolyte: (**A**) 70 mM Tris + 56 mM HCl + 140 mM NaCl (pH 7.5), (**B**) 57.5 mM Tris + 46 mM HCl + 115 mM NaCl (pH 7.5), PF solution: (A) Poly-dT_60_ at 0.46 mM, Poly-dT_5_ at 5.11 mM in 2.1 times diluted electrolyte. (**B**) Poly-dA_30_/dsDNA_dA-dT_ at 0.85 mM in 2.1 times diluted electrolyte. PF solution was introduced by hydrodynamic injection (1 bar, 4.5 min) into the capillary previously filled with the electrolyte. Abbreviations: Bta (*H*-β-(3-benzothienyl)-Ala-OH), CQ (chloroquine), dA (2′-deoxyadenosine), dsDNA_dT-dA_ (polydA-polydT hybrid double stranded DNA), dT (2′-deoxythymidine), ISP (isoprenaline), MDMA (methylenedioxymethamphetamine), 1-MT (1-methyltryptophan), 1-Nal (1-naphtylalanine), PL (propanolol), Qal(2′) (*H*-β-(2-quinolyl)-Ala-OH), TRA (tramadol). Trp (tryptophan), VPM (verapamil). Reprinted with permission from [66].

A study in 2017 systematically investigated the structural requirements for oligonucleotides as CS in PF-EKC [66]. Three observations were made. First, longer oligonucleotides increased analyte retention time and improved resolution. This is shown in Figure 4A where analytes were separated using two sets of poly-dT oligonucleotides. Second, single-stranded oligonucleotides were more effective than double-stranded oligonucleotides. This is shown in Figure 4B. Single-stranded oligonucleotides have certain structural features that are easily accessible to analytes, allowing selector-analyte interactions such as π-π stacking. Third, heteropolymeric oligonucleotides have better resolution than homopolymeric oligonucleotides, which was explained by the presence of multiple nucleotidic sites, allowing more interactions between the analyte and the selector.

### 2.3. Molecular Micelles

Molecular micelles contain individual surfactant monomers that are connected to one another. They were first used as BGS additives for EKC in the early 1990s [68,69,70] and the CS versions of these additives were demonstrated in the late 1990s [71]. Molecular micelles can be used at lower concentrations than conventional micelles, because the critical micelle concentration is zero. There were two amino acid-based molecular micelles, poly-(sodium undecyl-(l)-leucine-leucine) [72] and poly-(sodium undecyl-(l)-leucine-valine) [73], which were studied for the resolution of various racemates. 

## 3. Supramolecules

**Figure 5:**
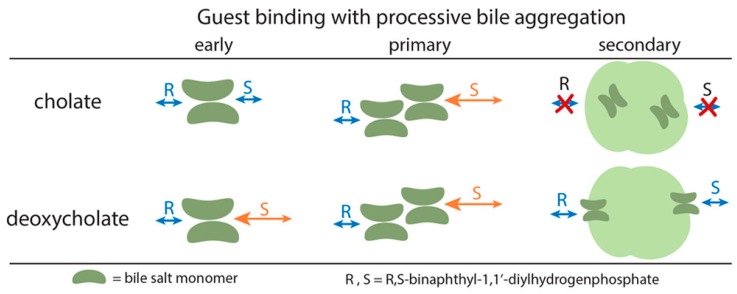
Schematic representation of guest binding with different bile salt aggregates. Pre-micellar or early are formed at low concentration, then primary and secondary aggregates are formed with increasing concentration of surfactant. Reprinted with permission from [75].

Micelles are aggregates of amphiphilic molecules such as surfactants. Chiral surfactants have been used as CS in EKC since the late 1980s [74]. For micelles formed from chiral surfactants, the fundamental basis for separation is the rapid exchange of enantiomers between the free and bound states of the corresponding micelles. There were two investigations that used bile salt micelles for EKC. First, in-depth knowledge on how bile salt chemistry leads to chiral selection was studied using cholate and deoxycholate bile salts [75]. Figure 5 shows the different aggregation architectures with increasing bile salt concentrations. It was found that the aggregation architectures play a significant role in recognition, where recognition was observed to disappear with larger aggregates (higher order or secondary micelles). Although NMR studies suggested the binding of 1,1′-binaphthyl-2,2′-diylhydrogen phosphate enantiomers to the secondary micelles of deoxycholate, EKC separation was not observed. Therefore, we suggest that future studies are required using a larger set of enantiomers to confirm the effect of higher order micelles. At lower concentration of bile salts, the smaller aggregates (pre-micellar and primary micelles) have recognition abilities because their binding sites are more accessible to the enantiomers. In another study, the separation mechanism of palonosetron hydrochloride with two chiral centers using sodium cholate was studied using a thermodynamic model [76]. The proposed model may be applicable to other chiral EKC systems. In our group, we are fundamentally developing a green, chiral electrochromatographic approach using surfactant-based pseudophases and chiral macromolecules in open-tube capillaries [77].

## 4. Low Molecular Weight Compounds

Chiral low molecular mass compounds can be elegantly used as a BGS additive that will interact with chiral target analytes in chiral EKC. Three classes of low molecular weight compounds were reported as CS. These are CS with a central ion (i.e., ligand exchangers [78,79], an ion-pair selector [80]), ILs [31,81,82] and a tetracycline antibiotic [83]. An IL implemented in a ligand exchanger is discussed under ILs. 

### 4.1. CSs with a Central Ion

Chiral ligand exchange (CLE) mechanism is based on the interchange between an analyte and a ligand, where the ligand is in a metal-ligand complex. The metal-ligand complex forms diastereomeric ternary metal-ligand-analyte complexes. The stability difference of the formed complexes has been considered as the basis for separation in CLE-EKC [84]. There have been claims that CLE-EKC is easy to optimise and manipulate the enantiomers’ migration order. There were ~1–3 papers a year from 2012–2016, but the use of CDs is still more popular. 

**Figure 6:**
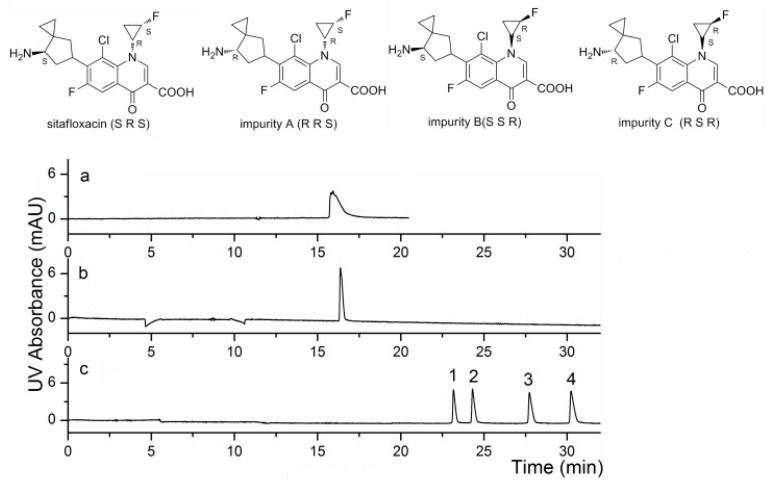
Electrochromatograms of sitafloxacin and its impurities under different separation conditions (**a**–**c**). Conditions: fused silica capillary, 60 cm (49.5 cm effective length) × 50 μm i.d.; sample injection: 6 s for 34 mbar; applied voltage, 15 kV; BGS: 15 mM dipotassium hydrogen phosphate (pH 4.2) containing (**a**) 20 mM γ-CD, (**b**) 10 mM Cu^2+^ and 10 mM d-Phe 10 mM, (**c**) 10 mM Cu^2+^, 10 mM d-Phe and 20 mM γ-CD. Peaks: 1 = impurity C (*R*,*S*,*R*), 2 = impurity B (*S*,*S*,*R*), 3 = impurity A (*R*,*R*,*S*), 4 = sitafloxacin (*S*,*R*,*S*). Reprinted with permission from [79].

The copper(II)-d-phenylalanine (Cu(II)-d-Phe) complex was used with γ-CD as dual CSs for the determination of sitafloxacin and its enantiomeric impurities [79]. Figure 6a–c show the electrochromatograms of sitafloxacin and its impurities using γ-CD, Cu(II)-d-Phe and γ-CD-Cu(II)-d-Phe, respectively. Under the optimised conditions, separation was not achieved using either γ-CD nor Cu(II)-d-Phe, and separation was only with the dual CSs. We suspected that the ligand exchanger served more as a chiral derivatising agent, where the resulting derivatives were separated by the CDs.

There have been reports on the improved chiral recognition in CLE-EKC by the addition of SDS micelles into the BGS [85]. Indeed, the use of SDS micelles was also critical in the separation of sitafloxacin and its impurities [79]. This was further validated in a report that used copper(II)-l-isoleucine complex with SDS micelles for the simultaneous analysis of ofloxacin enantiomers and six related impurities [78]. It was noted that without SDS, ofloxacin was only partially separated while the impurities were not separated at all. The separation of the achiral impurities is facilitated by micellar EKC mechanism [86].

Enantioseparation via ion-pairing with a chiral polydiol (di-*n*-butyl-l-tartrate)-boric acid complex (borospirane) in a nonaqueous BGS had been proposed in 2013 [87]. More recently, the analysis of seven different β-agonists was conducted using diacetone-d-mannitol-boric acid complex as the ion-pairing CS in methanol [80]. The low relative permittivity values in methanol provided an environment to achieve ion-pairing interactions [87]. Cationic enantiomers form diastereomeric ion pairs with the borospirane. The difference in their formation equilibrium forms the basis for their separation [88]. Others have considered that the enantioseparation mechanism with the use of borospiranes is CLE-EKC, with borate anion instead of a metal ion as the central ion [85]. Since cationic analytes without diol groups cannot replace the ligand and form a complex with boric acid, the ion-pairing is the correct mechanism for enantioseparation.

### 4.2. ILs

The utility of chiral monomeric and polymeric (micelles) ILs as CS was first recognised in 2006 [89]. Chiral ILs are organic salts that exist as liquid at room temperature. They consist of an organic cation and an organic or inorganic anion. Either the cation or the anion must be chiral to be considered a chiral IL. We note that an achiral IL derivatised β-CD was reported (see Section 2.1.1.2) in 2018. During this review period, amino acid or amide based chiral ILs were mainly used in conjunction with a macromolecule (mostly a CD) [31,60,82,90,91,92,93]. In general, chiral separations were obtained only with both IL and macromolecule in the BGS. For example, the IL tetrabutylammonium l-argininate (TBA^+^) was used with β-CD for the separation of different phenetylamine enantiomers [82]. It is likely that the synergy of the two CS was based the competition between TBA^+^ and analytes for inclusion into β-CD. The adsorption of TBA^+^ to the capillary surface altered the EOF that could have also affected the separation. Meanwhile, (R)-*N*,*N*,*N*-trimethyl-2-aminobutanol-bis(trifluoro-methanesulfonyl)imidate) in conjunction with γ-CD also improved separations in EKC [91].

A series of 1-alkyl-3-methylimidazolium l-prolinate ([C_n_mim][Pro], where n = 4, 8, 12) were recently characterised [81]. The [C_4_mim][Pro] was successfully used as ligand in CLE-EKC with copper as the central metal ion for the separation of d,l-tyrosine and d,l-tryptophan enantiomers. Enantioseparation also was affected by the chain length of the IL, with n = 8 or 12 giving lower performance. It was noted that an anodic EOF was observed at higher concentrations of the longer chain [C_12_mim][Pro] in the BGS. This suggested molecular aggregation of this IL at the fused silica capillary wall. However, the experiments conducted were limited to electrokinetic separations with the cathode at the detector end. The counter-EOF (anode at the detector end) and evaluation of micellar EKC separations with [C_12_mim][Pro] are suggested in the future.

### 4.3. Small Molecule Antiobiotic

Doxycycline is a common antibiotic that is already available in generic drug formulations. Recently, it was nicely explored as a new CS in EKC [83]. This was perhaps motivated by the several stereogenic centers and functional groups in its structure. Baseline separations were achieved in the analysis of eight chiral drugs and two 2,4-dinitrobenzoylamino acids under optimised conditions. Doxycycline can also exist as a cation in neutral or slightly acidic media. Thus, chiral recognition of the anionic analytes tested may also be facilitated by electrostatic interactions.

## 5. Molecular Modelling and NMR Studies

Molecular modelling is a technique that can provide a 3D docking picture of the analyte-CS/other molecule complexes. Molecular modelling can help the researcher understand the different steps involved in the molecular recognition process, the non-bonding interactions involved between the analyte and the CS and the binding energies for their association. During this review period, ten studies utilised molecular modelling, with the intention of understanding chiral recognition mechanism and elution migration order [22,32,35,44,60,62,72,73,94,95]. Molecular modelling has also been utilised to design new CSs [40].

In the case of molecular micelles in Section 2.3, their recognition mechanism was investigated using molecular modelling [72,73]. The nature of the polar heads affects the separations in CE. Both molecular micelles have pockets for binding sites, with the former and latter having six and four pockets, respectively. Test molecules bind preferentially to a pocket that hides the non-polar atoms from the solvent. Modelling results imply that stereoselective hydrogen bonding plays an important role in recognising enantiomers. Future studies are geared towards the generation of a model to predict the type of polar head appropriate for the separation.

It is important to understand the interactions occurring between the CS and the analyte. From this information, enantioselectivity and elution order can be easily predicted. Moreover, the classes of analytes that can be separated by a specific CS could be predicted as well. Nuclear magnetic resonance (NMR) studies can be used in conjunction with molecular modelling to understand chiral recognition. One-dimensional proton NMR (^1^H-NMR) spectroscopy allows to probe these interactions by looking at differences in chemical shifts. In other cases, the information obtained from ^1^H-NMR spectra is not enough to explain the observed behaviours. Nuclear Overhauser Effect spectroscopy (NOESY) or rotating frame Overhauser effect spectroscopy (ROESY) overcomes this limitation of ^1^H-NMR by giving information about the proximity between two atoms. ROESY is however preferred over NOESY because no signal enhancements may result from NOESY experiments [96]. 2D NOESY experiments are sufficient when specific interactions between analyte and CS are not required. During this review period, there were six studies that utilised either ^1^H- NMR, NOESY or ROESY techniques. These NMR techniques were used mainly to determine the structure of the formed analyte-CS/other molecule complexes [35,36,39,75,94,97]. NMR was used to investigate the CE behaviour of bile salts [75], determine host:guest stoichiometry [97] or understand enantiomer migration order in CE [35,94].

Molecular modeling and NMR was used to study chiral recognition with a BGS that contained γ-CD and SDS for the separation of ambrisentan [97]. Guided by the results of NMR spectroscopy using a solution of γ-CD:SDS:analyte at the ratio 1:2:1 and molecular dynamics simulations, it was claimed that the formation of ternary complexes involving γ-CD, SDS monomer and the analyte was important. However, with the high concentration of SDS in the BGS used (i.e., 100 mM), the formation of stable γ-CD-SDS complexes is expected to be favoured over the formation of the proposed ternary complexes. Under the conditions reported for separation, the γ-CD:SDS molar ratio of 1:10 (10 mM γ-CD, 100 mM SDS) is much higher compared to the ratio of 1:2 used in the NMR studies for the ternary complexes. Nevertheless, interesting studies on the complexation involving CDs, typical surfactants used in MEKC and targeted racemates should be pursued.

## 6. Applications to Real Samples

There were approximately 20 applications of the chiral EKC methods for real world analysis. An obvious utility of chiral EKC using is in pharmaceutical drug analysis. An important application of chiral separation methods is in the analysis of enantiomeric impurities. The differences in potencies and pharmacologic properties of drug substances make enantomeric impurity analysis vital. There were five studies that used chiral EKC for optical impurity analyses [19,24,30,34,54]. Derivatised β-CDs such as carboxymethyl-β-CD [24], sulfobutyl ether-β-CD [30,54], derivatized γ-CDs such as (2-hydroxypropyl)-γ-cyclodextrin [34] and dual chiral selectors such as α-CD with tetramethyl-ammonium-L-arginine [19] and were reported in the optical impurity analysis of various drugs in a synthetic mixture. Octa(6-*O*-sulfo)-γ-CD was used in the analysis of different pharmaceutical forms of pindolol, which is formulated as a racemic mixture [41]. Two groups worked independently on the separation of ofloxacin and related substances [33,78]. One group used copper(II)-L-isoleucine complex as CS [78], and the other used various β-CD derivatives, namely, carboxymethyl-β-CD and sulfated-β-CD [33]. Sodium carboxymethyl-β-CD was used in the analysis of betaxolol HCl and propranolol HCl in an inter-instrument reproducibility study that is required in method transfer [47]. A short end injection CE method was developed for anti-Alzheimer’s and antifungal drugs using sulfobutyl ether-β-CD as CS [95]. Meanwhile, the IL tetrabutylammonium L-glutaminate and hydroxypropyl-β-CD were used for the analysis of corynoxine and corynoxine B enantiomers in the stems of *Uncaria rhynchophylla* and its formulations [31]. Under the optimised conditions, both corynoxine and corynoxine B were enriched and separated, with good sensitivity. 

Carboxymethyl-β-CD was used for the analysis of *R*-(+)-higenamine in *Nelumbins plumula* powder [26]. Heptakis (2,6-di-*O*-methyl)-β-CD was used in a MEKC analysis of theanine and catechins in different green tea variants [25]. L-Theanine, the naturally occurring isomer in tea, can racemise into d-theanine. This may be used as a sample stability marker for green tea. Under their optimised conditions, theanine and the catechins were analysed simultaneously. Highly sulfated-γ-CD was used in the analysis of methadone, its metabolites and romifidine [49]. Ion-pairing chiral EKC was applied for the analysis of clenbuterol in a pharmaceutical solution containing also amboroxol. [80]. Finally, the dual CS β-CD and hydroxypropyl-β-CD was used in the analysis of D-glutamate and D-aspartate in rice wine [53]. These d-amino acids may serve as biomarkers of rice wine quality.

Environmental analysis is a potential application of chiral EKC, e.g., for monitioring or enantiomeric transformations under different environmental conditions. Chiral pollutants that occur at low levels in waters samples were enriched by solid-phase extraction (SPE) prior to EKC in two different studies [37,43]. In particular, SPE and chiral EKC analysis using a modified β-CD showed good precision, linearity, accuracy, low limits of quantitation (LOQ) and high sample recovery for the analysis of phenoxy acid herbicides enantiomers from river samples and wastewater treatment plant effluents [37]. LOQs were between 0.4 and 14.3 μg/L while recoveries ranged from 78.3 to 107.5%.

**Figure 7:**
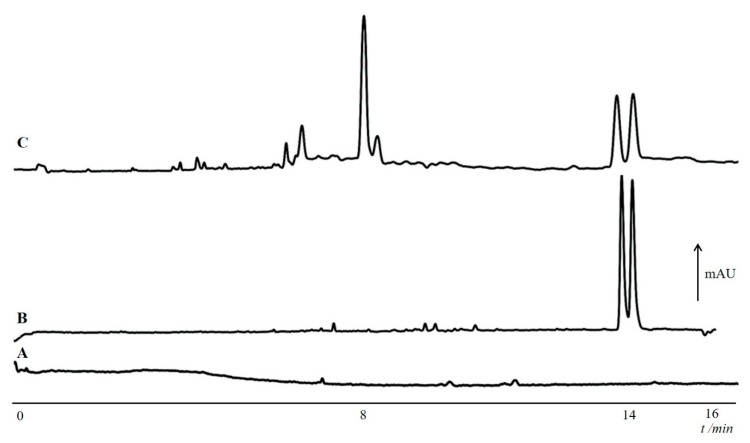
Sample electrochromatograms of (**A**) blank EBC, (**B**) an EBC spiked with 2.5 μg/mL methadone and (**C**) EBC sample of a patient under MMT. Conditions: uncoated fused silica capillary, 50 cm (41.5 cm effective length) × 50 μm i.d.; capillary temperature: 15 °C; sample injection: 40 s at 15 kV after preliminary pressure injection of water (1 s at 50 mbar); applied voltage: +25 kV; BGS: 150 mM phosphoric acid-TEA (pH 2.5) containing 30% methanol and 0.8% (*w*/*v*) carboxymethyl-β-CD. Reprinted with permission from [48].

Analysis of chiral compounds in biological samples is another potential application of chiral EKC. In one report, chiral EKC analysis using quaternary ammonium β-CD was developed for d-aspartic acid and d-glutamic acid in single neuron cells [46]. This was motivated by studies that reported the physiological importance of d-amino acids in living organisms. Note that the L-form of amino acids was historically believed to be the only important form. On-line sample concentration or stacking in CE was also exploited because of the low amino acid concentrations in the samples [98]. This decreased limits of detection to the sub-picomolar concentrations. Stacking in chiral EKC with carboxymethyl-β-CD was also developed for the analysis of methadone in exhaled breath condensate (EBC) [48]. The method was applied to patients undergoing methadone maintenance therapy (MMT). Figure 7 shows representative electrochromatograms from the analysis of blank EBC sample (Figure 7A), spiked EBC sample (Figure 7B), and EBC sample from patient undergoing MMT (Figure 7C). The fit-for-use of the method is clear from the data shown in Figure 7C, where methadone enantiomers in the patient sample were clearly found (see Figure 7C). However, the need to separate the enantiomers for the analysis of methadone in these samples is unclear. Maltodextrin was used as CS in the analysis of tramadol and methadone in human urine and serum samples [59]. The method was suitable for use as the analysis was achieved without environmental interferences.

## 7. Conclusions

EKC will remain a popular choice for research in chiral separation due to its high separation power and inherent greenness (the small amounts of organic solvents used and scale). However, publications relating to real sample applications of new CSs and methodologies only comprise 36% of all papers covered. This could be because of the intellectual property protection for the chiral compounds being developed in the chemical and pharmaceutical industries. This prevents authors from publishing research data in traditional and open-access journals. Not surprisingly, applications published during 2017–2018 were mainly on pharmaceutical drug analysis, but there were also interesting reports in environmental water, biological and food/wine sample analysis. 

We covered in this review the developments on the use and preparation of CSs for EKC. Macromolecular CSs, especially the CDs were the most common because they are easily accessible with acceptable purity and synthesis of their derivatives had become easier. There was a notable number of 14 papers that used molecular modelling and/or NMR to understand chiral recognition of CSs. Several papers have demonstrated the use two CS in EKC, for improved chiral separation. One CS is a macromolecule while the other could be an ionic liquid or ligand exchanger. It is noteworthy that the use of ionic liquids in synergism with CDs to expand the separation ability of CDs is becoming a popular area of study. In addition, while SDS micelles are known to improve chiral recognition of CSs such as CDs and ligand exchangers, a study on liposomes as an achiral additive has been shown to provide a similar effect for a CD.
